# Hyperpolarized ^13^C-pyruvate MRI detects real-time metabolic flux in prostate cancer metastases to bone and liver: a clinical feasibility study

**DOI:** 10.1038/s41391-019-0180-z

**Published:** 2019-11-04

**Authors:** Hsin-Yu Chen, Rahul Aggarwal, Robert A. Bok, Michael A. Ohliger, Zi Zhu, Philip Lee, Jeremy W. Gordon, Mark van Criekinge, Lucas Carvajal, James B. Slater, Peder E. Z. Larson, Eric J. Small, John Kurhanewicz, Daniel B. Vigneron

**Affiliations:** 1 Department of Radiology and Biomedical Imaging, University of California, San Francisco, CA USA; 2 Department of Medicine, University of California, San Francisco, CA USA

**Keywords:** Cancer metabolism, Prostate cancer, Translational research

## Abstract

**Background:**

Hyperpolarized (HP) ^13^C-pyruvate MRI is a stable-isotope molecular imaging modality that provides real-time assessment of the rate of metabolism through glycolytic pathways in human prostate cancer. Heretofore this imaging modality has been successfully utilized in prostate cancer only in localized disease. This pilot clinical study investigated the feasibility and imaging performance of HP ^13^C-pyruvate MR metabolic imaging in prostate cancer patients with metastases to the bone and/or viscera.

**Methods:**

Six patients who had metastatic castration-resistant prostate cancer were recruited. Carbon-13 MR examination were conducted on a clinical 3T MRI following injection of 250 mM hyperpolarized ^13^C-pyruvate, where pyruvate-to-lactate conversion rate (*k*_PL_) was calculated. Paired metastatic tumor biopsy was performed with histopathological and RNA-seq analyses.

**Results:**

We observed a high rate of glycolytic metabolism in prostate cancer metastases, with a mean *k*_PL_ value of 0.020 ± 0.006 (s^−1^) and 0.026 ± 0.000 (s^−1^) in bone (*N* = 4) and liver (*N* = 2) metastases, respectively. Overall, high *k*_PL_ showed concordance with biopsy-confirmed high-grade prostate cancer including neuroendocrine differentiation in one case. Interval decrease of *k*_PL_ from 0.026 at baseline to 0.015 (s^−1^) was observed in a liver metastasis 2 months after the initiation of taxane plus platinum chemotherapy. RNA-seq found higher levels of the lactate dehydrogenase isoform A (Ldha,15.7 ± 0.7) expression relative to the dominant isoform of pyruvate dehydrogenase (Pdha1, 12.8 ± 0.9).

**Conclusions:**

HP ^13^C-pyruvate MRI can detect real-time glycolytic metabolism within prostate cancer metastases, and can measure changes in quantitative *k*_PL_ values following treatment response at early time points. This first feasibility study supports future clinical studies of HP ^13^C-pyruvate MRI in the setting of advanced prostate cancer.

## Introduction

Metastatic castration-resistant prostate cancer (mCRPC) is the most lethal form of the disease, accounting for 31,000 deaths/year in the United States [1]. More than 90% of patients with mCRPC develop osseous metastases and nearly half have bone as the only site of the disease [2, 3]. Visceral metastases occur in 10–15% of mCRPC patients and are associated with high disease burden and poor prognosis [4, 5]. Despite the emergence of multiple therapies that have been shown to prolong overall survival, including androgen pathway inhibitors, immunotherapy, radiopharmaceuticals, and chemotherapeutics, there is an unmet need for novel therapies to further improve treatment outcomes [3, 6, 7].

A limitation to the development of novel systemic therapies in mCRPC, especially with bone predominance without measurable disease by conventional imaging criteria, is the lack of validated imaging biomarkers to provide real-time response monitoring. Automated bone indices of radionuclide bone scans have not been sufficiently prospectively validated, and provide minimal information with respect to direct tumor metabolic activity. Also, changes in bone scintigraphy with response to therapy can be slow to occur and are complicated by flare phenomena and differences in uptake between sclerotic versus lytic lesions [8]. Newer PET analogs, including agents targeting prostate-specific membrane antigen, have shown promise as a diagnostic tool, but have limited and conflicting data to support their use to monitor therapeutic response and resistance [9, 10].

**Fig. 1:**
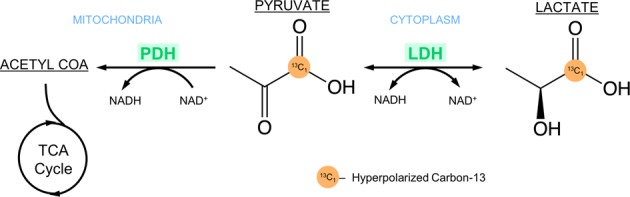
An illustration of LDH-mediated aerobic glycolysis and relevant metabolic pathways

Hyperpolarized ^13^C MRI (HP ^13^C MRI) is a stable-isotope molecular imaging approach that probes pyruvate-to-lactate metabolism mediated by the upregulation of LDH enzymatic activity in cancer due to the Warburg effect [11–14] (Fig. 1). High glycolytic activity and rapid pyruvate-to-lactate conversion are signatures of aggressive cancer [13, 15, 16]. There is also a broad consensus that the pharmacologic action of chemotherapy is tightly coupled with metabolic pathways, and responses to chemotherapy might be reflected as modulations of cancer metabolism [17–19]. Heretofore HP ^13^C-pyruvate MRI has been successfully utilized in prostate cancer only in localized disease. This imaging modality has been used to detect metabolic responses to chemohormonal therapy in primary prostate cancer [20], at earlier time points than conventional multiparametric MRI.

In the current pilot imaging study, we aimed to broaden the scope of HP ^13^C-pyruvate MRI to the metastatic CRPC setting, with direct visualization of skeletal and visceral metastases, in order to provide real-time assessment of tumor metabolism and metabolic response to therapy.

## Methods

### Patient selection

Key eligibility criteria included histologic evidence of prostate cancer, progressive mCRPC by PCWG2 criteria [7], ECOG performance status of 0 or 1, and adequate end organ function. All patients underwent restaging CT and bone scans prior to enrollment, and had at least one identified lesion amenable to HP ^13^C MRI. Patient recruitment and HP ^13^C-pyruvate studies were conducted in compliance with an IRB-approved protocol (NCT02911467), and all patients provided written informed consent.

### HP ^13^C patient MRI studies

GMP [1^−13^C]pyruvic acid (Sigma-Aldrich Isotec, Miamisburg OH) was prepared and loaded in pharmacy kits in accordance with the IRB- and FDA IND-approved stable-isotope manufacturing process. The pyruvic acid was polarized in a 5T SPINLab (GE Healthcare, Chicago IL) clinical trial polarizer for 2.5–3 h. Dissolutions yielded 237 ± 10 mM sterile pyruvate with 37.1 ± 3.2% polarization, 0.6 ± 0.4 μM residual radical and 31.0 ± 0.6 °C temperature, 7.5 ± 0.3 pH, 63 ± 4 s dissolution-to-injection time. A pharmacist oversaw the automatic quality control and integrity of the sterilization filter, and released the dose for injection once sterility and safety criteria were met [21, 22].

All studies were conducted on a clinical 3T MRI (MR750, GE Healthcare) equipped with multinuclear spectroscopy capabilities. A custom surface coil with figure-eight configuration was applied for both ^13^C transmit and receive. A 16-channel abdominal array (GE Healthcare) was used for proton imaging.

Follow-up ^13^C-pyruvate MRI was optional after the initiation of systemic therapy for the treatment of mCRPC.

### Data acquisition and analysis

The HP-^13^C acquisition was conducted using a 2D dynamic MR spectroscopic imaging pulse sequence with a slice-selective spectral-spatial excitation, followed by phase-encode and echo-planar spectroscopic imaging readout [11]. Pulse sequence parameters were as follows: 130 ms/3.5 ms TR/TE, 2–3 cm slice thickness, 1.2–1.5 cm in plane spatial and 3 s temporal resolutions, 60 s acquisition window, 545 Hz bandwidth, constant flip angle through time with pyruvate 10°, and lactate 20°. Scan started 5 s following the end of the injection. Patients were asked to hold their breath as long as possible, after which they were instructed to breathe gently and resume breath holding as tolerated. Conventional proton T_1_-weighted spoiled gradient-echo (TR/TE = 4.3 ms/1.9 ms) images were acquired for anatomic reference. Dynamic HP ^13^C MRI datasets were processed by applying even–odd lobe phasing, B_0_-shift correction, tensor-low-rank signal enhancement [23], spectral baseline correction [24], followed by a phase-sensitive peak quantification. The pyruvate-to-lactate conversion rate, *k*_PL_, was evaluated using an inputless single-compartment two-site exchange model [25], and the value reported was the maximum over ROI of the lesion identified on proton MRI. Total carbon signal-to-noise ratio (SNR) was reported as summed SNR of ^13^C-labeled tracers averaged over time. The image processing tools are located under—SIVIC Image Processing/Display: https://sourceforge.net/projects/sivic, Hyperpolarized MRI Toolbox: https://github.com/LarsonLab/hyperpolarized-mri-toolbox.

### Metastatic tumor biopsy acquisition and analysis

CT-guided metastatic tumor biopsies following HP MRI acquisition were obtained in five out of the six patients enrolled in the study (Table 1). Tumor biopsies were obtained for both fresh frozen processing and formalin fixed paraffin embedded (FFPE) processing. FFPE tissues were used for histologic diagnosis, while frozen tissue underwent Laser Capture Microdissection for RNA-seq profiling as previously described [6]. Expression levels reported as log(1 + (TPM × 10^6^)). Processed RNA-seq data are located in the Supplementary Materials.

**Table 1:** A summary of clinically relevant information from each patient

Patient	Age^a^	Target metastatic site	Gleason score^b^	Serum PSA (ng/ml)^a^	Metastatic tumor biopsy pathology	Most recent prior systemic therapy
1	75	Left iliac wing	4 + 5	171.7	Adenocarcinoma + Small cell neuroendocrine carcinoma (SCNC)	Enzalutamide + Investigational agent
2	57	Liver	4 + 4	Baseline: 38 Follow-up: 13.4	High-grade adenocarcinoma	Investigational agent
3	83	Rib	4 + 4	89.6	No biopsy performed	Enzalutamide
4	72	Right posterior ilium	4 + 5	89.2	High-grade adenocarcinoma	Docetaxel + Ribociclib
5	70	Left posterior ilium	4 + 5	1482	High-grade adenocarcinoma	Docetaxel
6	82	Liver	4 + 3	1439	High-grade adenocarcinoma	Docetaxel + Carboplatin

^a^At study entry

^b^At initial diagnosis

## Results

### Patient characteristics

Six patients were enrolled in this pilot feasibility study. The baseline characteristics of the patients are shown in Table 1. All patients had progressive mCRPC at study entry. Five of the patients underwent CT-guided metastatic tumor biopsy of the target lesions following completion of baseline ^13^C-pyruvate MRI. No adverse events were reported throughout this study.

### HP ^13^C-pyruvate MRI detects high *k*_PL_ in bone and liver metastases

**Fig. 2:**
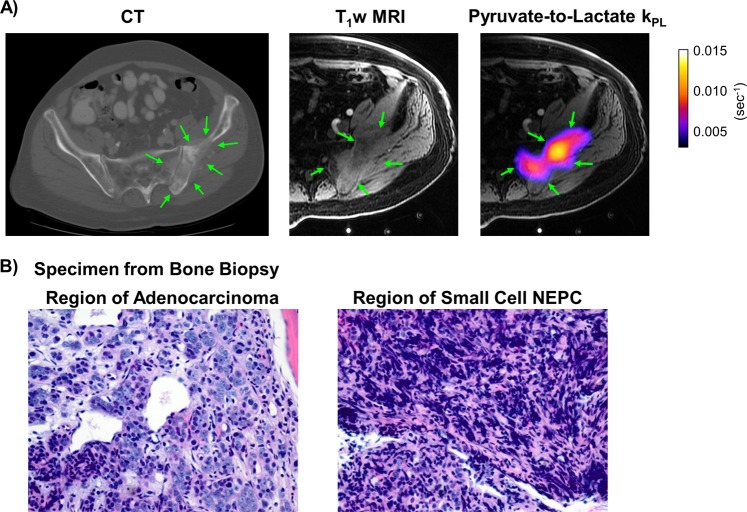
**a** Patient 1 (75 years old) was diagnosed with metastatic castration-resistant prostate cancer with several large osteoblastic lesions throughout the left hemipelvis and involving left femur. CT identified a relatively osteolytic lesion in left ilium (Green arrows), measuring 9.9 × 4.1 cm. The lesion was infiltrative, causing destruction of the bone cortex and extension into the surrounding soft tissues. T1-weighted (T_1_w) spoiled gradient-echo MRI was used to target the same lesion observed on CT for the HP ^13^C MR acquisition. Regions of high pyruvate-to-lactate conversion rate (*k*_PL_) correlated with the osseous lesion on CT and hypointensity on T_1_w MRI. *k*_PL_ was estimated 0.013 (s^−1^). **b** The paired bone biopsy demonstrated discrete regions of adenocarcinoma and treatment-emergent small cell neuroendocrine differentiation

The rate of conversion of pyruvate to lactate (*k*_PL_) from target lesions in each patient is listed in Table 2. There was high *k*_PL_ in both bone and liver metastases, with mean *k*_PL_ of (0.020 ± 0.006 s^−1^) and (0.026 ± 0.000 s^−1^), respectively [26]. Regions of high *k*_PL_ were consistent with CT and MRI radiographic findings of metastatic disease presence, as shown in the representative *k*_PL_ image overlays for the target lesions (Fig. 2a, Supplementary Figs. 1–4).

**Table 2:** Findings from HP ^13^C MRI including *k*_PL_, and RNA expression of key genes

Patient	*k*_PL_ of target lesion (s^−1^)	Ldha/Pdha1 expression (in log)	SNR tCarb
1	0.013	15.4/12.3	290.3 ± 248.5
2	Baseline: 0.026 Follow-up^a^: 0.015	16.2/13.8	Baseline: 89.7 ± 40.9 Follow-up^a^: 77.7
3	0.017	Not acquired	131.4 ± 10.2
4	0.026	14.7/11.6	27.4 ± 9.9
5	0.023	16.4/12.6	19.5 ± 3.6
6	0.025	15.6/13.7	88.2 ± 26.1

SNR tcarb: summed SNR of ^13^C-labeled tracers averaged over time

^a^Follow-up was 2 months after initiation of carboplatin + docetaxel

The liver mass of patient six showed considerable intratumoral heterogeneity (Supplementary Fig. 4b). Maximum *k*_PL_ = 0.025 (s^−1^) was found in viable tumor, whereas *k*_PL_ = 0.004 (s^−1^) was observed in a necrotic-appearing region identified both on CT and the delayed phase of contrast T_1_-weighted images.

Table 2 also summarizes the total carbon SNR for each study. The total carbon SNR was 117 ± 126 in bone metastases (*N* = 4), and 85 ± 7 between liver involvements (*N* = 2). In general, the SNR in all cases was adequate for reliable *k*_PL_ fitting (standard error metric *σ*_kPL_ = 0.005 ± 0.003) [27].

In all five patients with paired ^13^C-pyruvate MRI and CT-guided biopsy of the target lesions, the histological evidence of metastatic prostate cancer was detected. In four of the five cases, the histology demonstrated poorly differentiated adenocarcinoma. In one patient (Patient 1), the paired metastatic tumor biopsy demonstrated discrete regions of adenocarcinoma and treatment-emergent small cell neuroendocrine differentiation (Fig. 2b) [6].

Higher levels of gene expression of the lactate dehydrogenase isoform A (Ldha,15.7 ± 0.7) relative to the dominant isoform of pyruvate dehydrogenase (Pdha1, 12.8 ± 0.9) were detected on RNA-seq of the target metastatic biopsies (Table 2), consistent with enhanced aerobic glycolysis detected in the rate of conversion of pyruvate to lactate on HP ^13^C MRI. No significant difference in Ldha or Pdha1 expression was observed in the patients imaged in this study compared with a previously published cohort of metastases from 200 men with mCRPC (Ldha: 15.1 ± 1.1, *p* > 0.17; Pdha1: 12.0 ± 0.9, *p* > 0.06, Wilcoxon ranked sum test) [6].

### HP ^13^C MRI detected a metabolic rate decrease in a metastasis following chemotherapy

**Fig. 3:**
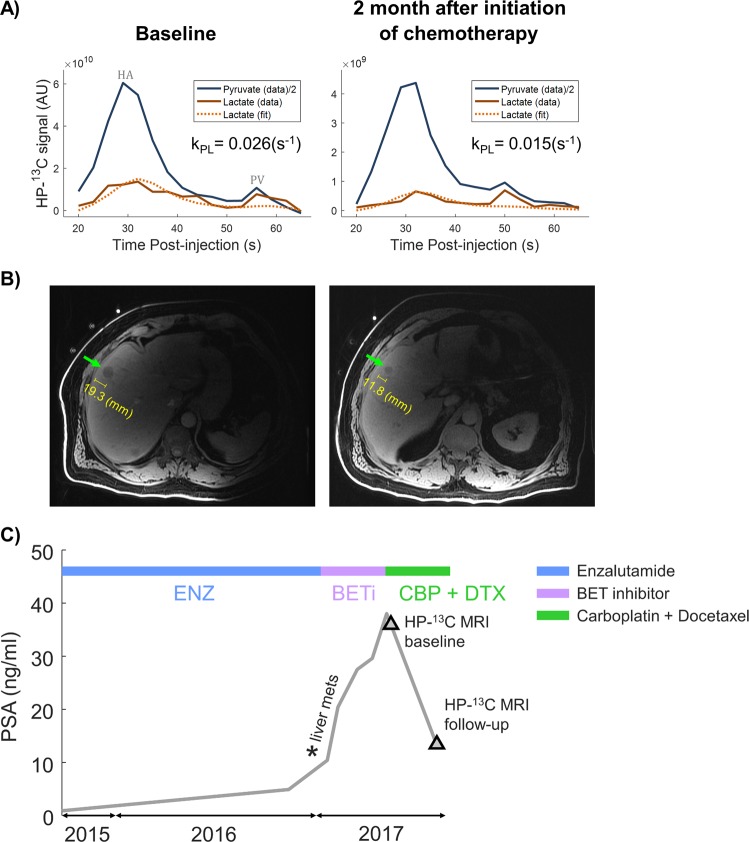
Patient 2 (57 years old) was diagnosed with CRPC that metastasized to liver. The patient was previously treated with enzalutamide and an investigational agent (BET inhibitor, phase I) with clinical progression. Chemotherapy of carboplatin and docetaxel started ~1 month post baseline HP ^13^C scan, and follow-up was 2 months after initiation of therapy. **a** A decrease in pyruvate-to-lactate conversion rate *k*_PL_ was observed from 0.026 to 0.015 (s^−1^) after 2 months of chemotherapy. Note the increase in pyruvate and lactate at 50–60 s post injection. Most likely this is predominately due to vascular contributions coming from intestines. **b** Follow-up 2 months after initiation of therapy found a decrease in lesion size (19.3–11.8 mm) indicating therapeutic response based on RECIST criteria. **c** In addition, serum PSA decreased from 38–13.4 ng/ml also indicating therapeutic response. HA arterial phase, PV portal venous phase

Patient 2 had mCRPC with liver metastases and low serum PSA level. Carboplatin + docetaxel chemotherapy was started 24 days after the baseline HP ^13^C MRI study (Fig. 3c). Follow-up HP MRI study 62 days after the initiation of treatment demonstrated a 42% decrease in pyruvate-to-lactate conversion rate *k*_PL_, from 0.026 to 0.015 s^−1^, in the target liver lesion (Fig. 3a). This was accompanied by interval decrease of the lesion size (Fig. 3b, 19.3–11.8 mm, 39%) based on RECIST 1.1 criteria, along with serum PSA decline of >50% from baseline (38–13.4 ng/ml), consistent with systemic treatment response.

## Discussion

This work reports the results of the first-ever pilot imaging study of prostate cancer metastases using HP ^13^C-pyruvate MRI. This study demonstrates the feasibility of detecting real-time metabolic activity of metastases and capture therapeutic response with this emerging stable-isotope molecular imaging method. Correlation with paired metastatic biopsy demonstrated high-grade prostate adenocarcinoma, including in one case, evidence of neuroendocrine differentiation.

The high pyruvate-to-lactate conversion rate, *k*_PL_, via upregulated LDH activity in cancer, known as Warburg effect, reflects cancer aggressiveness, and decrease in *k*_PL_ can reflect therapeutic response [14, 20]. Overall, the pyruvate-to-lactate conversion rate *k*_PL_ found in bone (0.020 ± 0.006 s^−1^) and liver (0.026 ± 0.000 s^−1^) lesions was either higher than or comparable with that of high-grade primary prostate cancer (0.013 ± 0.003 s^−1^) in a cohort imaged prior to radical prostatectomy with whole mount section pathologic correlation [26]. These high *k*_PL_ values were correlated with the metastatic biopsy findings of high-grade adenocarcinoma or mixed high-grade adenocarcinoma and small cell neuroendocrine phenotypes in the patients studied in this report.

Elevated Ldha expression in prostate cancer is known to be associated with aggressive phenotypes and resistant to therapy [28–30]. The high Ldha expression relative to Pdha1 in this study was consistent with a larger published mCRPC cohort [6], reflective of enhanced aerobic glycolysis, whereas in normal prostate epithelial cells the glucose metabolism favors oxidative phosphorylation, and Pdha1 expression should predominate (Fig. 1). This suggested that the metabolic features observed in this study using HP ^13^C-pyruvate MRI could potentially serve as a representative cross-section of a much larger patient population with different metastatic sites and types of cell morphology.

A previous study in primary prostate cancer indicated that *k*_PL_ reflected early response and resistance to androgen pathway inhibition [20]. In this communication we observed a correlation between decreased *k*_PL_ and clinical response to the combination of platinum plus taxane chemotherapy in a patient with mCRPC. Although preliminary, these findings suggest that imaging metabolic signatures using HP ^13^C-pyruvate MR could potentially report responses to a broader range of oncogenic pathway inhibition and drug targets, and may be less susceptible to the upregulation of membrane protein expression secondary to ADT [31, 32]. These data suggest that the prospective evaluation of HP ^13^C-pyruvate as a response biomarker in mCRPC patients treated with AR-targeting and cytotoxic chemotherapy is warranted.

Spatially, regions of high *k*_PL_ showed good alignment with radiographic findings of metastases using bone scan, CT, and proton MRI. Temporally, the time-to-peak of pyruvate bolus was 29 ± 3 s in pelvic bone cases, 23 s in the rib case, 34 ± 4 s among the liver cases. The bolus delivery timing was generally consistent with contrast CT/MRI [33], and are deemed reasonable in light of hemodynamic variations between subjects, and also the vitals of individual subject at the time of the scan. The inputless *k*_PL_ model applied in this study is relatively immune to variations in bolus characteristics [25].

Differential *k*_PL_ was observed between viable and necrotic-appearing regions of Patient 6’s liver lesion (Supplementary Fig. 4b). These findings are consistent with other emerging reports of intra- and inter-tumoral heterogeneity in mCRPC [34] and highlight the potential utility of this imaging tool to clarify tumor biology with real-time metabolic monitoring [34–37]. The *k*_PL_ heterogeneity between metastatic sites/individual patients and its biological underpinning calls for future investigation.

This study also demonstrated that this technology can provide quantitative metrics of the delivery/uptake of the injected hyperpolarized carbon isotope by measuring the total carbon SNR summing the ^13^C signal observed from the hyperpolarized pyruvate bolus and downstream metabolic products. Conceptually similar to SUV in PET, total carbon SNR is a metric of delivery and uptake. Of the pelvic bone involvements, patient 1, whose lesion appeared relatively more lytic on CT (Fig. 2a), had higher mean total SNR_Patient 1_ = 290 versus the other two cases (SNR_Patient 4_ = 27.4, SNR_Patient 5_ = 19.5) with more sclerotic appearances (Supplementary Figs. 1 and 3). This presents an intriguing concordance with PET literature in which osteolytic lesions have shown higher FDG uptake compared with osteoblastic ones [38–40] and glucose metabolism is known to be differently regulated in sclerotic versus lytic diseases [41].

Several key limitations should be identified for this pilot study. The correlation between metabolic biomarker *k*_PL_ and total carbon SNR is yet to be elucidated in the mCRPC setting. In addition, the test–retest repeatability data are also needed moving forward. While the total carbon SNR reports tracer pharmacokinetics at each metastatic site, its quantitative accuracy can be further enhanced using automatic B_1_ calibration and correction for QC parameters. This study utilized a 2D single-slice imaging strategy. Future advancement in array receiver hardware [42] and MR acquisition sequences [21, 43] will enable full 3D coverage of the abdomen/pelvis and seamless integration with standard-of-care restaging scans. Dissemination of this technology, in terms of infrastructure and instruments, requires a clinical ^13^C polarizer and specialized MRI hardware. The on-site pharmaceutical manufacturing follows the same standard as PET, allowing for shared facility [14]. These capabilities can readily be instated in high-volume tertiary centers who manages the majority of the advanced prostate cancer cohort. Overall, future developments are warranted to address the technical needs including hardware, image acquisition and quantitative analyses, and the clinical inquiries deserve to be powered by a larger cohort study.

These preliminary results highlight the future need to metabolically characterize lymphadenopathy using HP ^13^C-pyruvate MRI, as management of nodal disease could be essential in the realm of biochemically recurrent and oligometastatic PCa [44–47]. For these cohorts of patients, opportunities for curative treatment are more available, and clinical outcomes are generally better than those with bone involvement and thus higher disease burden. Such future studies could be enabled by the aforementioned technical advancements to achieve higher resolution and sensitivity, and new pharmacy QC procedures that reduce HP ^13^C-pyruvate time-to-injection and thereby improving SNR [14].

## Conclusions

This pilot study evaluated the safety and feasibility to conduct HP ^13^C MRI studies of patients with metastatic prostate cancer to the skeleton and viscera, which represents the most advanced and lethal form of the disease. Methods were examined and established for instrumentation setup, pharmacy manufacturing, image acquisition, and quantitative analysis. Safety was demonstrated and highly upregulated pyruvate-to-lactate conversion *k*_PL_ was observed on aggressive osseous and hepatic metastases. Interval decrease of *k*_PL_ was found for one patient receiving combination chemotherapy, in concordance with conventional clinical biochemical and imaging biomarkers. These findings warrant further development and investigation of HP ^13^C-pyruvate MRI in a larger prospective group of men with metastatic CRPC.

## Supplementary information

Supplemental Material-RNA-seq Data

Supplemental Figures
