# Supplementary material for: Hyperpolarized 13C-pyruvate MRI detects real-time metabolic flux in prostate cancer metastases to bone and liver: a clinical feasibility study
Source: Prostate Cancer Prostatic Dis. 2019 Nov 4;23(2):269–76. doi: 10.1038/s41391-019-0180-z (PMC7196510; doi:10.1038/s41391-019-0180-z)
Supplement: Supplementary file 2 — Supplemental Figures [file 41391_2019_180_MOESM2_ESM.pdf]

Supplemental Figures

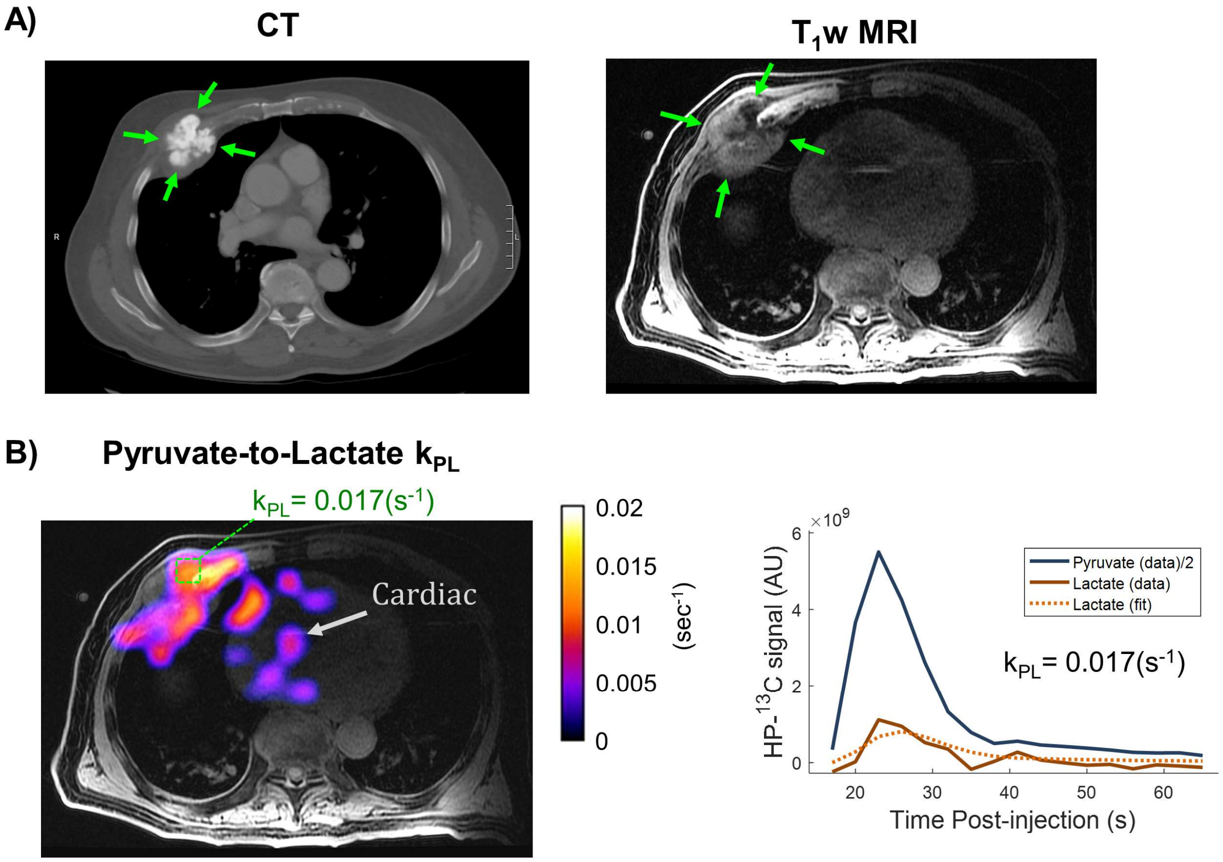

Supplemental Figure 1. Patient 3 was 83-year-old who was diagnosed with Gleason 4+4 prostate cancer with progressive mCRPC status post multiple prior lines of therapy (Table 1). **(A)** CT images identified an osseous lesion on right lateral 4th rib, measuring 6.5x4.8cm, with soft tissue components. Center of the lesion appeared sclerotic on CT and hypoenhancing on T_1w MRI. **(B)** k_{PL} of 0.017(s^{-1}) was measured in the rib lesion. The tumor mass extended posteriorly along the 4th rib, spatially agreeing with high k_{PL} regions.

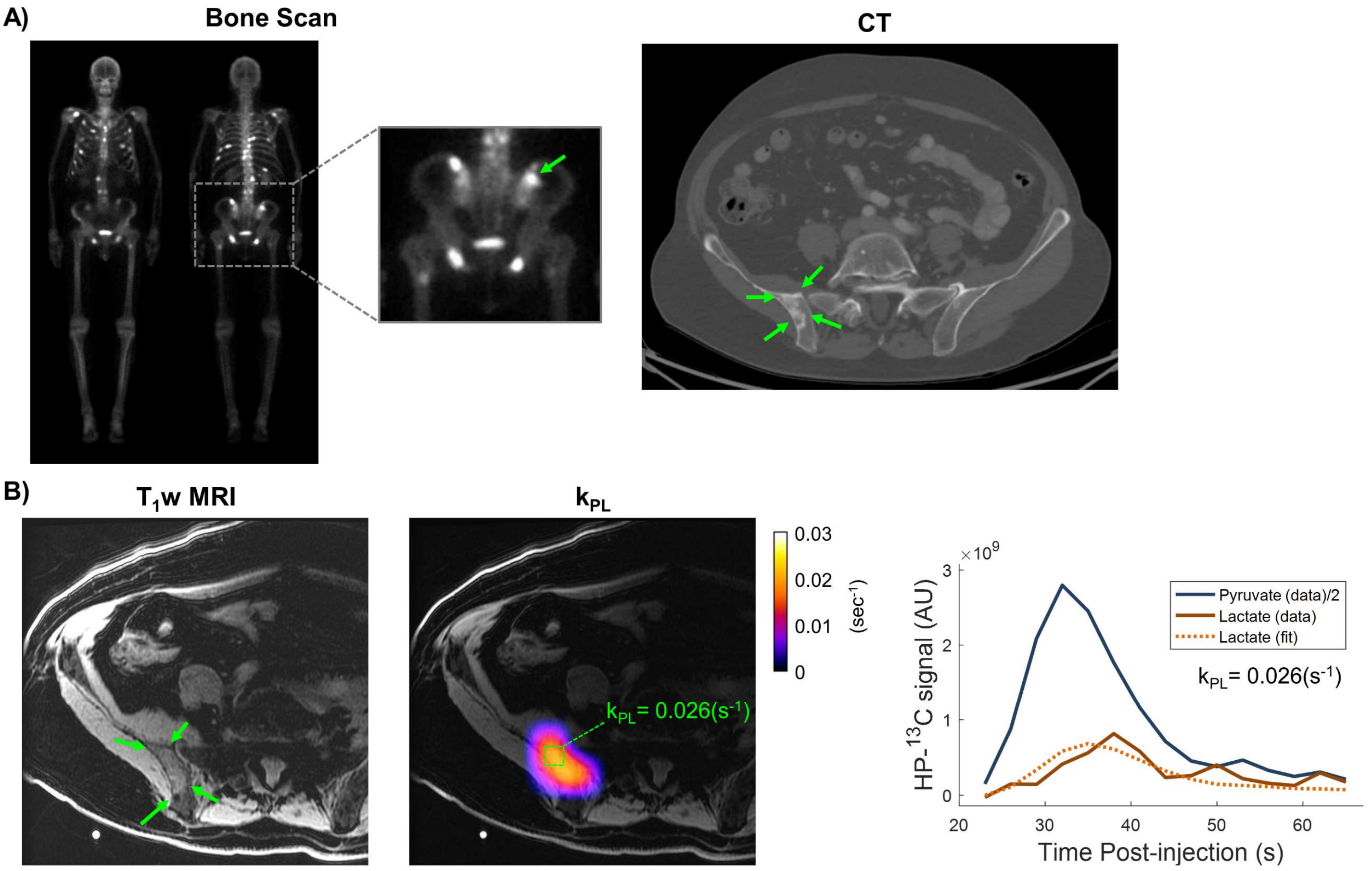

Supplemental Figure 2. Patient 4 was a 72-year-old individual with Gleason 4+5, cT1c prostate adenocarcinoma at diagnosis. **(A)** A bone scan revealed extensive disease in rib cage, spine and pelvis. CT identified an osteoblastic lesion at right iliac crest (2.3x1.4 cm) with some cortical erosion. **(B)** k_{PL} in the osseous lesion was calculated 0.026(s^{-1}). CT-guided bone biopsy confirmed adenocarcinoma consistent with mCRPC, and PSA was 89.2 ng/ml at the time of HP study.

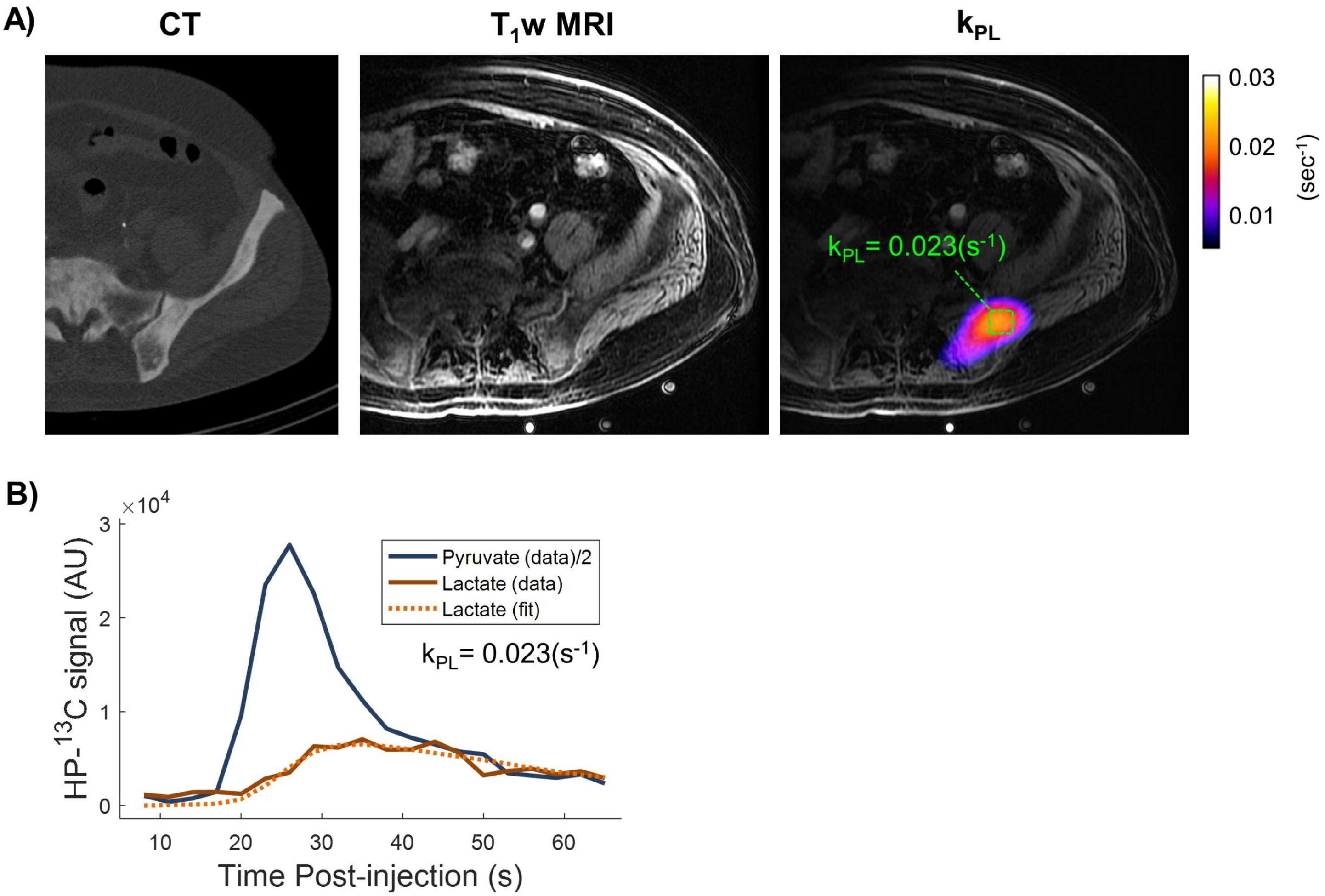

Supplemental Figure 3. Patient 5 was 70-year-old diagnosed with Gleason 4+5 prostate cancer. Bone metastases involving the ribs, spine and pelvis was found at the time of diagnosis. Restaging bone scan and CT identified extensive sclerotic metastases throughout axial into the appendicular skeleton, involving essentially all visualized bone in the chest, abdomen and pelvis. **(A)** HP ^{13}C study targeted a diffuse sclerotic lesion adjoining the left iliac spine. Deep-bone biopsy found adenocarcinoma consistent with mCRPC. **(B)** k_{PL} was measured 0.023(s^{-1}) at the lesion, and PSA was 1482 (ng/ml) at the time of the exam.

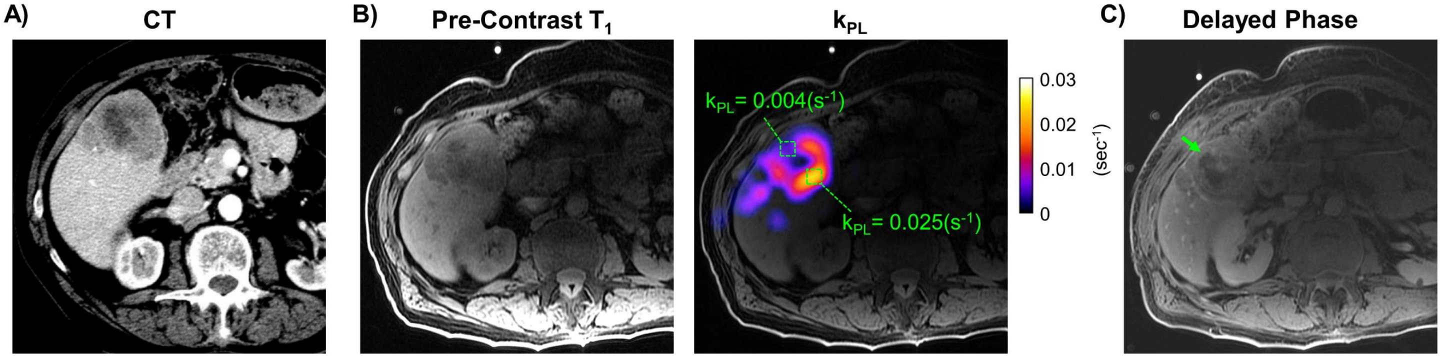

Supplemental Figure 4. Patient 6 was an 83-year-old patient with mCRPC including large liver metastases. **(A)** CT identified a large lesion in segment 5 measuring 5.5x5.1cm, with centrally hypoenhancing/necrotic-appearing region measuring 2.8x2.3cm. The HP ^{13}C study **(B)** estimated k_{PL} at 0.025(s^{-1}). Note region of low k_{PL} in HP MRI correlated with the necrotic-appearing region in the **(C)** delayed phase of contrast imaging. Fine needle aspiration to the lesion of interest found adenocarcinoma consistent with mCRPC.
